# DpaA Detaches Braun’s Lipoprotein from Peptidoglycan

**DOI:** 10.1128/mBio.00836-21

**Published:** 2021-05-04

**Authors:** Matthias Winkle, Víctor M. Hernández-Rocamora, Karthik Pullela, Emily C. A. Goodall, Alessandra M. Martorana, Joe Gray, Ian R. Henderson, Alessandra Polissi, Waldemar Vollmer

**Affiliations:** a Centre for Bacterial Cell Biology, Biosciences Institute, Newcastle University, Newcastle upon Tyne, United Kingdom; b Institute for Molecular Bioscience, The University of Queensland, Brisbane, Queensland, Australia; c Dipartimento di Scienze Farmacologiche e Biomolecolari, Università degli Studi di Milano, Milan, Italy; d Biosciences Institute, Newcastle University, Newcastle upon Tyne, United Kingdom; University of Utah

**Keywords:** *Escherichia coli*, cell envelope, peptidoglycan, Braun’s lipoprotein (Lpp), periplasm, amidase, lipoproteins

## Abstract

Gram-negative bacteria have a unique cell envelope with a lipopolysaccharide-containing outer membrane that is tightly connected to a thin layer of peptidoglycan. The tight connection between the outer membrane and peptidoglycan is needed to maintain the outer membrane as an impermeable barrier for many toxic molecules and antibiotics. *Enterobacteriaceae* such as Escherichia coli covalently attach the abundant outer membrane-anchored lipoprotein Lpp (Braun’s lipoprotein) to tripeptides in peptidoglycan, mediated by the transpeptidases LdtA, LdtB, and LdtC. LdtD and LdtE are members of the same family of ld-transpeptidases but they catalyze a different reaction, the formation of 3-3 cross-links in the peptidoglycan. The function of the sixth homologue in E. coli, LdtF, remains unclear, although it has been shown to become essential in cells with inhibited lipopolysaccharide export to the outer membrane. We now show that LdtF hydrolyzes the Lpp-peptidoglycan linkage, detaching Lpp from peptidoglycan, and have renamed LdtF to peptidoglycan *meso*-diaminopimelic acid protein amidase A (DpaA). We show that the detachment of Lpp from peptidoglycan is beneficial for the cell under certain stress conditions and that the deletion of *dpaA* allows frequent transposon inactivation in the *lapB* (*yciM*) gene, whose product downregulates lipopolysaccharide biosynthesis. DpaA-like proteins have characteristic sequence motifs and are present in many Gram-negative bacteria, of which some have no Lpp, raising the possibility that DpaA has other substrates in these species. Overall, our data show that the Lpp-peptidoglycan linkage in E. coli is more dynamic than previously appreciated.

## INTRODUCTION

Bacteria have to maintain the integrity of their complex cell envelope at all times when propagating in diverse and often adverse environments (1). The peptidoglycan (PG) layer surrounds the cytoplasmic membrane (CM) and protects the cell from rupture due to osmotic challenges (2, 3). Diderm (Gram-negative) bacteria protect themselves from antimicrobial compounds by surrounding their PG layer by a semipermeable outer membrane (OM). In Escherichia coli and related species, the PG and OM are tightly connected by multiple highly abundant proteins, such as Lpp (Braun’s lipoprotein), OmpA, and Pal (4–7). The cell must maintain these tight connections at all times and coordinate the expansion of the PG and OM during growth and cell division to avoid leaks in the OM, the loss of OM vesicles, or lysis due to an instable cell envelope (6, 8, 9).

Lpp is the most abundant protein in E. coli, with about 1 million copies per cell (5). In pioneering work in the 1970s, Volkmar Braun and coworkers identified Lpp not only as the first protein that is covalently attached to PG but also as the first protein with a lipid modification (4). The Lpp preprotein uses the Sec system for translocation through the CM, where it then matures by (i) the addition of a diacylglycerol to a cysteine residue near the N-terminus, (ii) the removal of the leader peptide, and (iii) the addition of a third acyl chain to the N-terminus (10). Mature Lpp is then transported through the periplasm and anchored to the inner leaflet of the OM by the localization of lipoprotein (Lol) transport system (11). About one-third of the protein molecules are covalently attached to PG via the ε-amino group of the C-terminal lysine, which is linked to the α-carboxylic group at the l-center of *meso*-diaminopimelic acid (*meso-*Dpm) at position 3 of a PG stem peptide, by the ld-transpeptidases (LDTs) LdtA, LdtB, and LdtC (4, 12, 13). About 5% of all PG subunits are attached to Lpp in exponentially growing cells and 15% in stationary phase (14).

In addition to stabilizing the cell envelope, Lpp helps to maintain a constant distance between the PG and OM (15, 16). Mutants lacking Lpp or LdtA, LdtB, and LdtC suffer from hypervesiculation, losing OM-derived vesicles filled with periplasmic content into the environment (9, 17, 18). These mutants become sensitive to sodium dodecyl sulfate (SDS) due to abnormally high OM permeability (6). Pathogenic E. coli and *Salmonella* mutants lacking Lpp are less virulent and unable to invade their host, and an Lpp-deficient uropathogenic E. coli strain is more susceptible to serum killing (19–21). In this strain, the attachment of Lpp to PG was crucial for the expression of capsular polysaccharide, illustrating another key role of PG-attached Lpp (21).

Given the important role of Lpp for cell envelope stability in E. coli, it was surprising that Lpp is present only in closely related *Gammaproteobacteria* (22). While another PG-attached lipoprotein has been described for *Pseudomonas* spp., the covalent attachment of PG to OM-localized proteins has not been studied in depth for PG of other diderm bacteria (23). This changed recently when two publications reported the covalent attachment of certain OM β-barrel proteins (OMPs) in a wide range of *Alphaproteobacteria* (24, 25). Additionally to OMPs, one of the studies identified the OM-anchored lipoprotein LimB attached to PG in Coxiella burnetii (25). Similar to Lpp in E. coli, these proteins are attached to the *meso-*Dpm residue in PG stem peptides by LDTs and are required for OM stability. In contrast to Lpp, OMPs were found to be attached to PG via N-terminal glycine or alanine residues. Many *Firmicutes* (Gram-positives) attach multiple cell wall proteins to PG using the enzyme sortase (26). These are often involved in interactions with host factors during bacterial infections and include, for example, protein A in Staphylococcus aureus or pilus subunits in Streptococcus pneumoniae (27, 28).

Although previous work identified several PG-attached proteins and two classes of enzymes for protein-PG attachment (LDTs and sortase), to date no enzyme for the opposite reaction, the detachment of proteins from PG, has been identified. Here, we show that an LDT homologue of previously unknown function (DpaA, formerly named LdtF or YafK) has an amidase activity hydrolyzing the bond between Lpp and PG in E. coli. We also report that the DpaA-mediated detachment of Lpp from PG helps the cell to cope with certain stress conditions and that DpaA-like enzymes are present in many diderm bacteria.

## RESULTS

### *dpaA* is genetically linked to PG and OM biogenesis.

**FIG 1:**
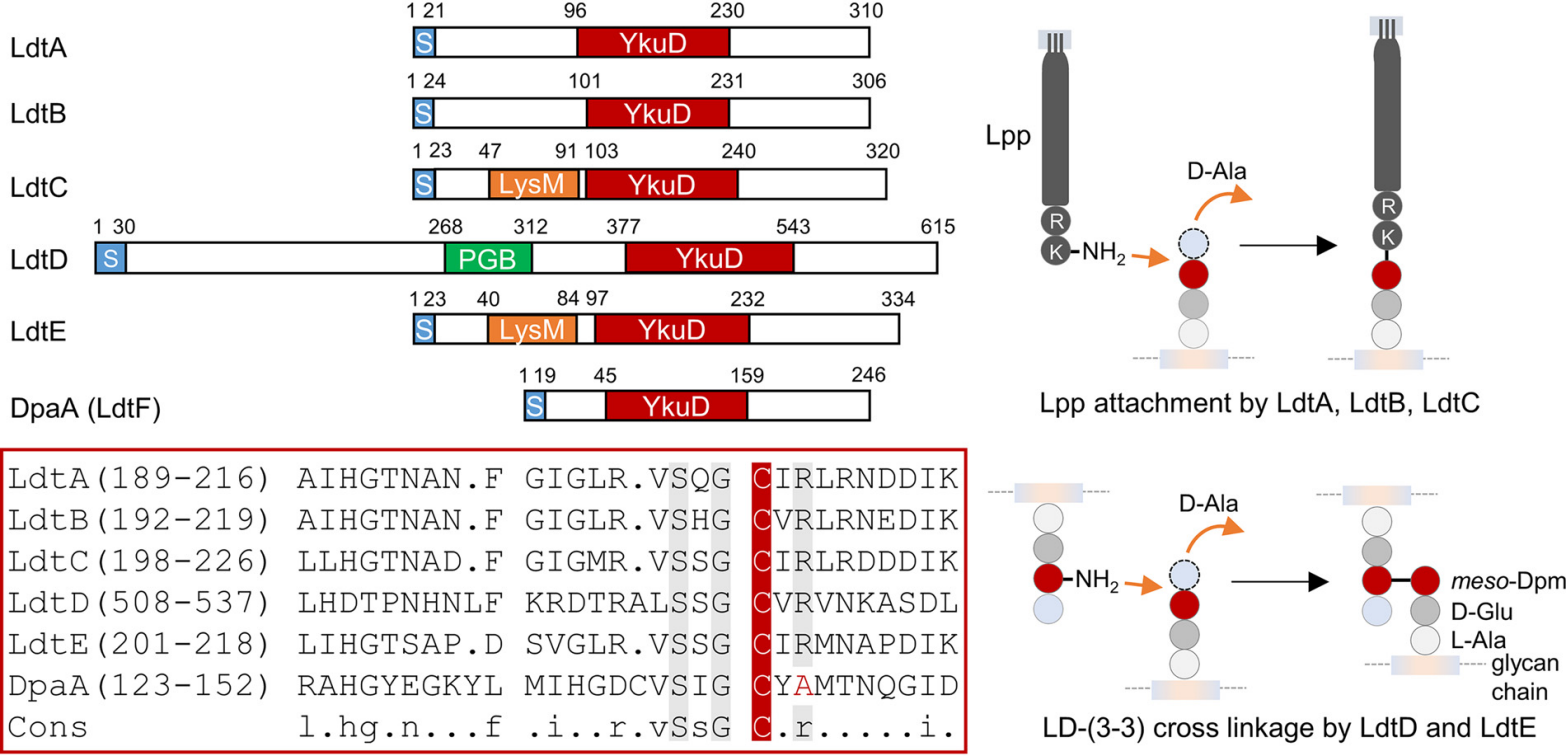
Overview of the different LDT family members. E. coli has six proteins with a YkuD (LDT) domain (top left). Protein regions: S, signal peptide; LysM and PGB, peptidoglycan-binding domains. The numbers indicate amino acid positions. Sequence alignment within the YkuD domain (bottom left) shows the active site cysteine (C, in red) and other conserved amino acid residues in gray. DpaA (LdtF) misses a conserved arginine (R), and its function is unknown. On the right side, it can be seen that LdtA, LdtB, and LdtC attach Lpp to *meso-*Dpm in PG stem peptides. LdtD and presumably LdtE form 3-3 cross-links in PG. l-Ala, l-alanine; d-Glu, d-glutamic acid; d-Ala, d-alanine; *meso*-Dpm, *meso*-diaminopimelic acid.

In E. coli, all six proteins with a YkuD domain (Fig. 1) become important under cell envelope stress conditions. LdtA, LdtB, and LdtC facilitate cell envelope stability by covalently attaching the OM-anchored lipoprotein Lpp to PG (13). LdtD forms 3-3 cross-links in PG to repair the PG layer upon defective OM assembly (29). LdtE appears to form 3-3 cross-links when cells enter the stationary phase of growth, although its activity has not been demonstrated with the purified enzyme. The function of the last protein, LdtF (now DpaA), remained unknown. *dpaA* and *ldtE* are regulated by RpoS and maximally expressed in stationary-phase cells. The expression of *dpaA*, but not *ldtE*, slightly increases upon lipopolysaccharide (LPS) stress (29). Genetic depletion of the essential LPS export gene *lptC* causes lysis in cells lacking *dpaA* (29), but these show morphological defects even in the presence of inducer (arabinose), and the lysis of *lptC*-depleted Δ*dpaA* cells can be prevented by the additional deletion of a novel, cell envelope stress-induced amidase activator gene, *actS* (30, 31). None of these phenotypes are observed for the other *ldt* mutants, suggesting that DpaA has roles different from (or additional to) those of the other LDTs.

**FIG 2:**
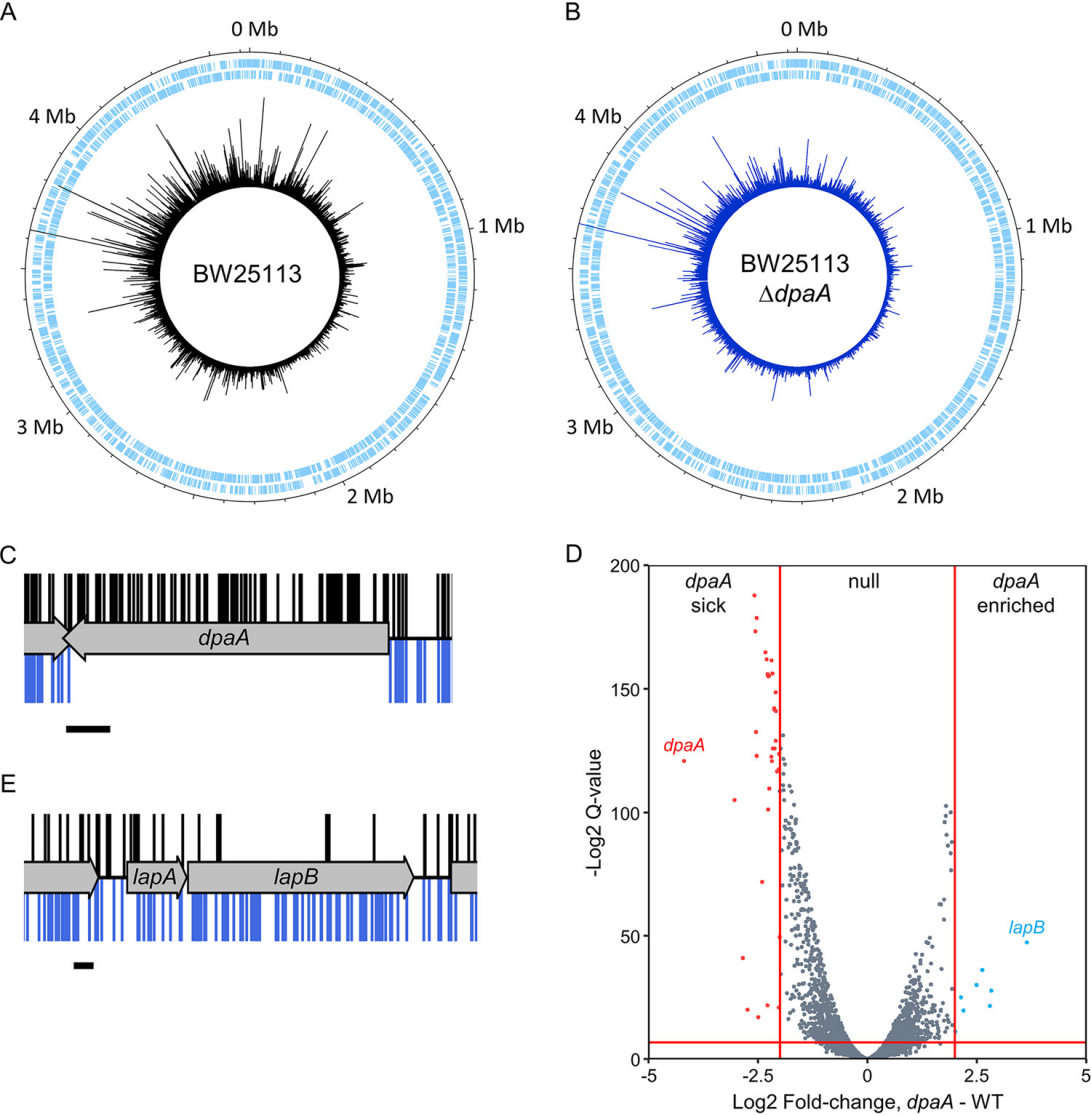
Comparison of the read depth between genes in each transposon library. (A and B) Genome maps representing the transposon mutant libraries constructed in BW25113 (A) and BW25113 Δ*dpaA* (B). The sense and antisense coding sequences are displayed in the two outermost tracks, respectively, in pale blue. The position and frequency of transposon insertion events around the BW25113 reference genome are represented by the peaks in the innermost track, plotted using DNAPlotter with a window size of 1 and a step size of 1. (C) Insertion profile of *dpaA*. (D) Volcano plot showing a comparison of the relative mutant abundance between libraries. Genes with a >2-fold change in read abundance and with a *q* value of <0.01 (see Materials and Methods) are colored; these thresholds are represented as solid red lines. WT, wild type. (E) Insertion profile of the *lapAB* operon. In panels C and E, the transposon insertion sites identified in the control library are displayed above the gene track (black) and transposon insertion events in the *dpaA* library are shown below the gene track (blue). The insertion plot is capped at a read frequency of 1. Scale bar = 100 bp.

To better understand the role of DpaA, we first performed genome-wide transposon insertion analysis (TraDIS) of the *dpaA* mutant. We constructed two libraries of transposon mutants by electroporation of a mini-Tn*5* transposon into a Δ*dpaA* strain and into the BW25113 parent strain as a control. To identify the transposon insertion sites, we sequenced the transposon-genomic DNA junction and mapped the data to the BW25113 reference genome as previously described (32). We identified 744,751 and 546,518 unique insertion sites for *dpaA* and the control library, respectively, and these are distributed around the chromosome (Fig. 2A and B). There were no insertions within *dpaA* in the Δ*dpaA* library, confirming the library genotype (Fig. 2C). To identify mutants with a fitness defect, or advantage, in the *dpaA* gene deletion background, we used the BioTraDIS EdgeR analysis tool to compare the frequency of reads per gene between each library, using thresholds of >2-fold change and *q* value of <0.01. This analysis revealed relatively few genes with significantly more or fewer reads in the *dpaA* mutant than in the wild type (Fig. 2D; see also Table S1 in the supplemental material). The most obvious difference we observed in the *dpaA* library compared to the control was a 3.64-fold enrichment (*q* value = 5.97E−15) of reads within *lapB* (*yciM*) (Fig. 2D and E). There were insufficient insertions within adjacent *lapA* (*yciS*) gene to quantify the difference between the libraries. LapA and LapB are essential regulators of LPS biosynthesis, regulating the degradation of LpxC by the membrane-bound protease FtsH (33, 34), and LapA physically interacts with the elongasome (35). Hence, *dpaA* inactivation is linked to phenotypes related to both LPS synthesis (*lapB* inactivation) and LPS export (*lptC* depletion). To begin to understand these genetic interactions, we wanted to determine the enzymatic activity of DpaA.

10.1128/mBio.00836-21.6 TABLE S1 BioTraDIS analysis: log fold change and *q* value scores for genes with >50 reads Table S1, XLSX file, 0.4 MB. Copyright © 2021 Winkle et al. 2021 Winkle et al. https://creativecommons.org/licenses/by/4.0/ This content is distributed under the terms of the Creative Commons Attribution 4.0 International license.

### DpaA is not an LD-TPase.

We purified LdtD, LdtE, and DpaA and first tested for their ability to produce 3-3 cross-links in PG isolated from an E. coli strain lacking all 6 LDTs (BW25113Δ6LDT) under neutral (pH 7.5) or acidic (pH 5.0) conditions. The PG was digested with the muramidase cellosyl, and the resulting muropeptides were separated by high-performance liquid chromatography (HPLC) (Fig. S1A and B). LdtD produced muropeptides with 3-3 cross-links or tripeptides [TetraTri(3-3), TriTri(3-3), TetraTri, and Tri (Fig. S1C)], confirming ld-transpeptidase (ld-TPase) and ld-carboxypeptidase (ld-CPase) activities (29). LdtE generated TetraTri(3-3), consistent with the presence of this TPase product in the PG of BW25113Δ6LDT cells overexpressing *ldtE* and *dpaA* (36). LdtE was nearly inactive at pH 7.5 and did not show ld-CPase activity. DpaA did not show any ld-TPase or ld-CPase activity under any condition tested, despite a long incubation time (12 h). We noticed that DpaA lacked an active-site arginine residue conserved in other LDTs (Fig. 1), suggesting that DpaA is an inactive enzyme of the LDT family or has a different activity than the other LDTs.

10.1128/mBio.00836-21.1 FIG S1 Distinct activities of LdtD/LdtE and DpaA. PG from BW25113Δ6LDT was incubated with LdtD, LdtE, or DpaA at pH 5.0 (A) or pH 7.5 (B). The PG was then digested with cellosyl and the resulting muropeptides were separated by HPLC. The ld-TPase product TetraTri(3-3) was present in reactions with LdtD or LdtE but not DpaA or the control without enzyme. (C) Structures of the relevant muropeptides. (D and E) Tri-LysArg incubated with DpaA and DpaA(C143A) (D) and LdtD or LdtE (E). Muropeptides were reduced with sodium borohydride and separated by HPLC. G, *N*-acetylglucosamine; M(r), *N*-acetylmuramitol; l-Ala, l-alanine; d-Glu, d-glutamic acid; d-Ala, d-alanine; *meso*-Dpm, *meso*-diaminopimelic acid. Download FIG S1, TIF file, 1.1 MB Copyright © 2021 Winkle et al. 2021 Winkle et al. https://creativecommons.org/licenses/by/4.0/ This content is distributed under the terms of the Creative Commons Attribution 4.0 International license.

### DpaA releases Lpp from PG.

We reasoned that rather than forming ld-bonds, DpaA might hydrolyze bonds generated by other LDTs; however, in several experiments we could not detect any ld-endopeptidase or ld-carboxypeptidase activity (data not shown). Hence, we next tested if DpaA hydrolyzes the bond between the terminal l-lysine residue of Lpp and PG, which is formed by LdtA-C (13). We first used purified muropeptides containing the terminal two amino acids (lysine and arginine) from Lpp as artificial substrates. The protease pronase E used in PG purification removes contaminating proteins and most part of the covalently attached Lpp, with the exception of the C-terminal Lys-Arg dipeptide; the residual dipeptide is used in PG analysis to quantify the amount of PG-attached Lpp in strains (14).

**FIG 3:**
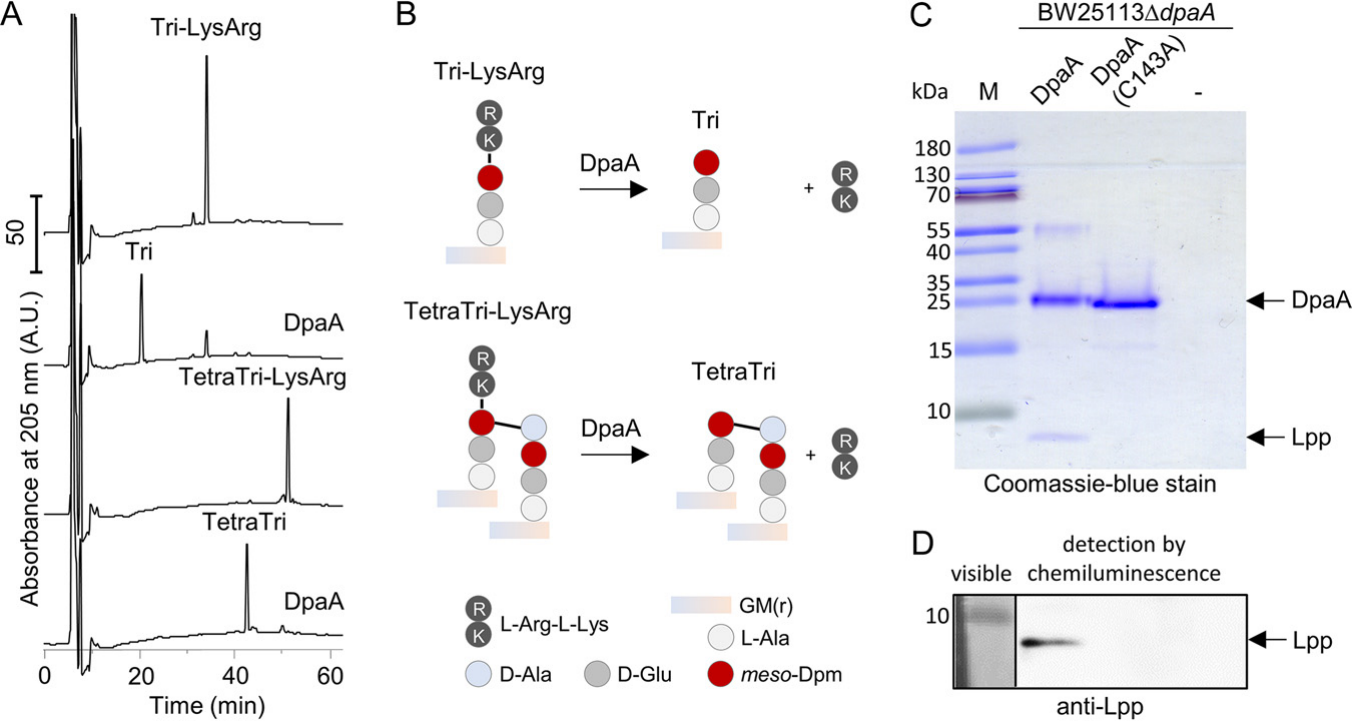
DpaA detaches Lpp from PG. (A) Activity against the muropeptides Tri-LysArg and TetraTri-LysArg. Muropeptides are the disaccharide peptide subunits released from PG by a muramidase. Tri-LysArg and TetraTri-LysArg (containing Lys-Arg from the C-terminus of Lpp) were incubated with DpaA, and the reaction products were reduced with sodium borohydride and separated by HPLC. DpaA hydrolyzed both substrates, releasing the muropeptides lacking l-Arg-l-Lys. The released dipeptide is missing in the chromatogram because it presumably coelutes early with the salts at ∼8 min. Table S2 shows the masses of the substrate and product muropeptides determined by mass spectrometry (MS). (B) Scheme of the reactions in panel A. Data in Fig. S1E show that LdtD and LdtE are not active against Tri-LysArg. Figure S2A and B show the inhibition of DpaA by copper. Muropeptide names are according to the work of Glauner et al. (14): Tri, disaccharide tripeptide; TetraTri, bis-disaccharide tetratripeptide. GM(r), *N-*acetylglucosamine-*N*-acetylmuramitol; l-Ala, l-alanine; l-Arg-l-Lys, l-arginyl-l-lysine dipeptide. (C and D) Lpp release assay. DpaA was incubated with PG sacculi from BW25113 *ΔdpaA* that were not treated with pronase E and hence contained PG-attached Lpp. Reaction mixtures were boiled and proteins were separated by SDS-PAGE, following by staining with Coomassie-blue (C) or Western blotting and detection of Lpp with specific antiserum (D). DpaA, but not the catalytically inactive DpaA(C143A), released Lpp from PG. M, protein size marker.

We incubated Tri-LysArg and TetraTri-LysArg with DpaA or the catalytically inactive DpaA(C143A) (with Ala replacing the active-site Cys residue) and analyzed the products by HPLC. DpaA, but not DpaA(C143A), converted both substrates to muropeptides lacking the Lys-Arg dipeptide (Tri or TetraTri) (Fig. 3A and Fig. S1D). We confirmed the mass of the substrate and product muropeptides by MS/MS analysis (Table S2). To test if this activity was specific for DpaA, we incubated Tri-LysArg with purified LdtD or LdtE and observed no activity with these LDTs (Fig. S1E).

10.1128/mBio.00836-21.7 TABLE S2 MS analysis of collected muropeptide fractions (related to Fig. 3) Table S2, PDF file, 0.04 MB. Copyright © 2021 Winkle et al. 2021 Winkle et al. https://creativecommons.org/licenses/by/4.0/ This content is distributed under the terms of the Creative Commons Attribution 4.0 International license.

10.1128/mBio.00836-21.2 FIG S2 DpaA is inhibited by copper and does not release d-Lys, d-Val, or d-Gln from PG. (A) PG from BW25113 *ΔdpaA* was incubated with DpaA in the presence of increasing concentrations of CuCl_2_. Muropeptides were generated, reduced, and separated by HPLC. DpaA activity is seen as the reduction in the Tri-LysArg peak, which decreased by copper in a concentration-dependent manner. Chromatographs were cropped above 250 mAU to better observe Tri-LysArg. (B) Quantification of the relative amounts of Tri-LysArg and Tri plotted against the concentration of CuCl_2_. The data are means ± variations of two independent experiments. (C) PG from BW25113Δ6LDT was incubated with LdtD and different d-amino acids, which were incorporated into position 4 of the stem peptides. An asterisk labels muropeptides with noncanonical d-amino acids. The PG was incubated with DpaA, followed by generation of muropeptides, reduction, and HPLC analysis. DpaA released glycine from PG but not the d-amino acids tested. Download FIG S2, TIF file, 1.3 MB. Copyright © 2021 Winkle et al. 2021 Winkle et al. https://creativecommons.org/licenses/by/4.0/ This content is distributed under the terms of the Creative Commons Attribution 4.0 International license.

LDTs are known to be inhibited by copper ions (37). To test if this was true for DpaA, we incubated PG from BW25113 *ΔdpaA* cells with DpaA in the presence of CuCl_2_, followed by quantification of Lys-Arg-containing muropeptides by HPLC. In the absence of copper, DpaA removed Tri-LysArg from the muropeptide profile, forming Tri, while the presence of 0.2 mM CuCl_2_ inhibited the reaction (Fig. S2A). We observed a half maximal inhibitory concentration (IC_50_) of ∼0.1 mM CuCl_2_ (Fig. S2B), which is approximately 6-fold-lower than that for LdtD (37).

In a different assay for DpaA activity, we incubated the protein with PG sacculi from BW25113 *ΔdpaA* cells that were not treated with pronase E and hence contained covalently attached full-length Lpp (rather than only the Lys-Arg dipeptide). Samples were centrifuged to separate soluble proteins from insoluble PG, and proteins present in the supernatant were analyzed by SDS-PAGE (Fig. 3C) and Western blotting with a specific antibody against Lpp (Fig. 3D). DpaA, but not inactive DpaA(C143A), was capable of releasing Lpp from PG, confirming the results obtained with the soluble muropeptides with Lys-Arg dipeptides. Altogether, our data prove that DpaA hydrolyzes the bond between *meso-*Dpm in PG and the C-terminal Lys residue of Lpp, releasing Lpp from the PG.

### DpaA also has CPase activity against glycine-containing muropeptides.

**FIG 4:**
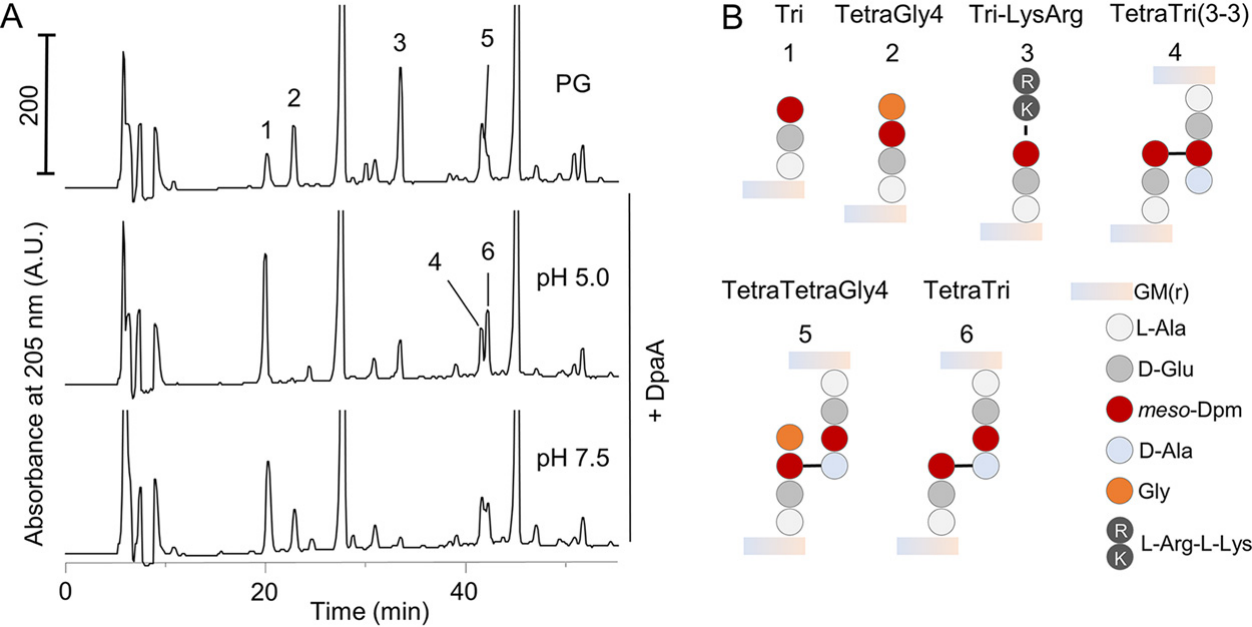
DpaA is active against TetraGly4 and TetraTetraGly4. (**A**) PG from an *araB*p*lptC* Δ*dpaA* (+arabinose) strain was incubated with DpaA at pH 5.0 or 7.5, followed by digestion with cellosyl and analysis of the muropeptide composition by HPLC. In addition to its expected decrease of Tri-LysArg, DpaA was also active against muropeptides containing glycine at position 4 (muropeptides 2 and 5). DpaA showed higher activity against TetraGly4 and TetraTetraGly4 at acidic pH. Chromatographs were cropped at 250 mAU to better observe the minor muropeptides. (B) Structures of the relevant muropeptide labeled in panel A. Figure S2C shows that DpaA is not active against muropeptides with noncanonical d-amino acids at position 4 of the PG stem peptide. Muropeptides names are according to the work of Glauner et al. (14).

When we further investigated the activity of DpaA. We found that it released Lys-Arg dipeptides from PG under acidic or neutral conditions (Fig. 4). In the course of these experiments, we noticed that DpaA also acted on rare peptides in PG with glycine (instead of d-Ala) at position 4, for example, TetraGly4 (Fig. 4B, peak 2) or its dimer version, TetraTetraGly4 (Fig. 4B, peak 5) (14). These structures were hydrolyzed by DpaA particularly under acidic conditions via a *meso-*Dpm-Gly CPase activity.

We next tested whether DpaA could remove other unusual amino acids present at the terminus of a tetrapeptide. For this, we used LdtD to exchange the terminal d-alanine residue by d-lysine, d-glutamine, d-valine, or glycine in PG from BW25113 Δ6LDT and tested whether these could be removed by DpaA (Fig. S2C). As before, DpaA was active against muropeptides with glycine but not against those with d-lysine, d-glutamine, or d-valine. Together, these data show that DpaA specifically removes Lys or Gly residues from position 4 of tetrapeptides, but not d-alanine or any of the other d-amino acids tested.

### DpaA is active in cells.

**FIG 5:**
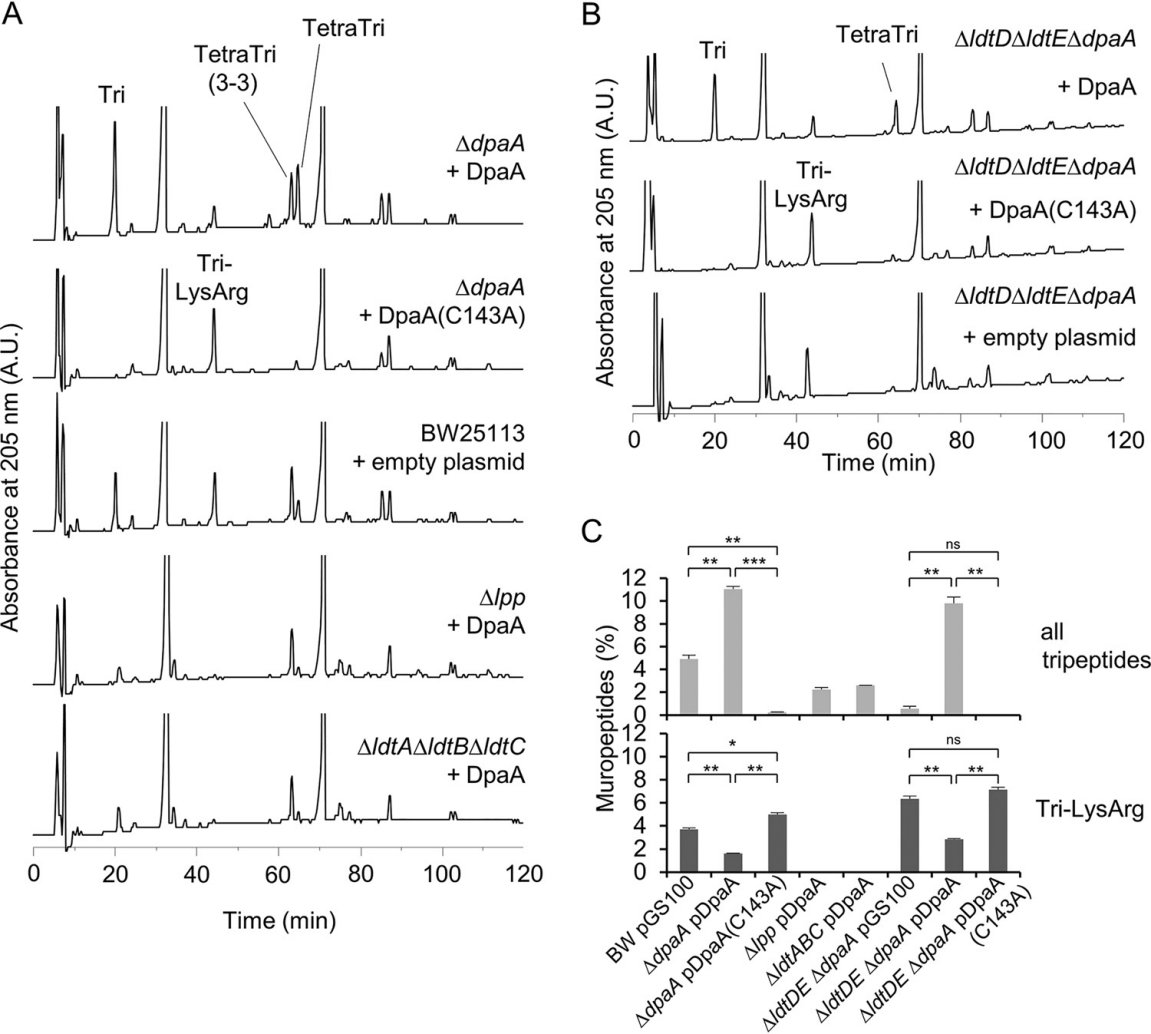
DpaA is active in cells. (A) The wild type (BW25113) or mutants with single or multiple gene deletions (BW25113 Δ*dpaA*, BW25113 Δ*lpp*, and BW25113 Δ*ldtA* Δ*ldtB* Δ*ldtC*) expressing DpaA or DpaA(C143A) from a plasmid, or carrying the empty plasmid, were grown in LB medium and harvested, and the muropeptide composition was determined. Only expression of active DpaA, and not the inactive DpaA(C143A), reduced the Tri-LysArg peak when present. (B) Muropeptide profiles of BW25113 Δ*ldtD* Δ*ldtE* Δ*dpaA* (lacking LDTs involved with 3-3 cross-link formation and DpaA) expressing DpaA, DpaA(C143A), or no DpaA version. Chromatographs were cropped above 250 mAU; the uncropped HPLC chromatographs of panels A and B are shown in Fig. S3. (C) Quantification of the total tripeptides and Tri-LysArg in the chromatograms shown in panels A and B. The values are means ± variations from two independent biological replicates, except BW25113 Δ*ldtA* Δ*ldtB* Δ*ldtC*/pDpaA, which was prepared once. Significance was measured by a two-tailed, homoscedastic *t* test. ns (not significant), *P* > 0.05; *, *P* ≤ 0.05; **, *P* ≤ 0.01; ***, *P* ≤ 0.001.

Our biochemical experiments provided strong evidence that DpaA is able to release Lpp from PG. To test whether this reaction can happen in cells, we ectopically expressed plasmid-borne *dpaA* or *dpaA(C143A)* in strains with deletions of the *dpaA*, *lpp*, or *ldtABC* genes and analyzed the PG composition of exponentially growing cells (Fig. 5A and B). Expression of DpaA in BW25113 *ΔdpaA* resulted in a 2-fold reduction of Tri-LysArg from 3.7% ± 0.1% to 1.6% ± 0.0%, compared to that for BW25113 expressing the empty plasmid (pGS100) (Fig. 5A and C). BW25113 *ΔdpaA* expressing catalytically inactive DpaA(C143A) had an increased level of Tri-LysArg compared to BW25113/pGS100, possibly due to the lack of DpaA activity. Tripeptides were increased in all samples with decreased Lys-Arg-containing muropeptides due to the expression of active DpaA; for example, BW25113 *ΔdpaA*/pDpaA contained 11.0% ± 0.3% tripeptides, compared to 4.9% ± 0.4% in BW25113/pGS100. This result suggests that cells counteract the high DpaA activity by increasing the activities of LdtA, LdtB and/or LdtC to maintain a sufficient amount of PG-attached Lpp.

10.1128/mBio.00836-21.3 FIG S3 DpaA is active in cells. Shown are full HPLC chromatograms of the cropped versions shown in Fig. 5 in the main text. Download FIG S3, TIF file, 0.9 MB. Copyright © 2021 Winkle et al. 2021 Winkle et al. https://creativecommons.org/licenses/by/4.0/ This content is distributed under the terms of the Creative Commons Attribution 4.0 International license.

The expression of DpaA had no significant effect on the PG composition in cells lacking Lpp (BW25113 *Δlpp*) or the Lpp-attaching enzymes (BW25113 *ΔldtA ΔldtB ΔldtC*), which, as expected, did not show detectable levels of Tri-LysArg and therefore lack the substrate of DpaA (Fig. 5A). To test whether LdtD or LdtE affects the activity of DpaA, we also analyzed the PG composition of BW25113 *ΔldtD ΔldtE ΔdpaA* cells containing pDpaA, pDpaA(C143A), or pGS100 (Fig. 5B). As expected, the PG of these cells did not contain 3-3 cross-links. The level of Tri-LysArg was higher in strains lacking *ldtD* and *ldtE*, presumably because LdtA, LdtB and LdtC compete with LdtD and LdtE for tetrapeptide donors in PG. However, the expression of active DpaA correlated with a reduction in Tri-LysArg and an increase in tripeptides in PG from BW25113 and BW25113 *ΔldtD ΔldtE ΔdpaA*, showing that the absence of LdtD and LdtE does not affect DpaA. Altogether, these data verify that DpaA detaches Lpp from PG in cells.

### DpaA contributes to mecillinam resistance.

**FIG 6:**
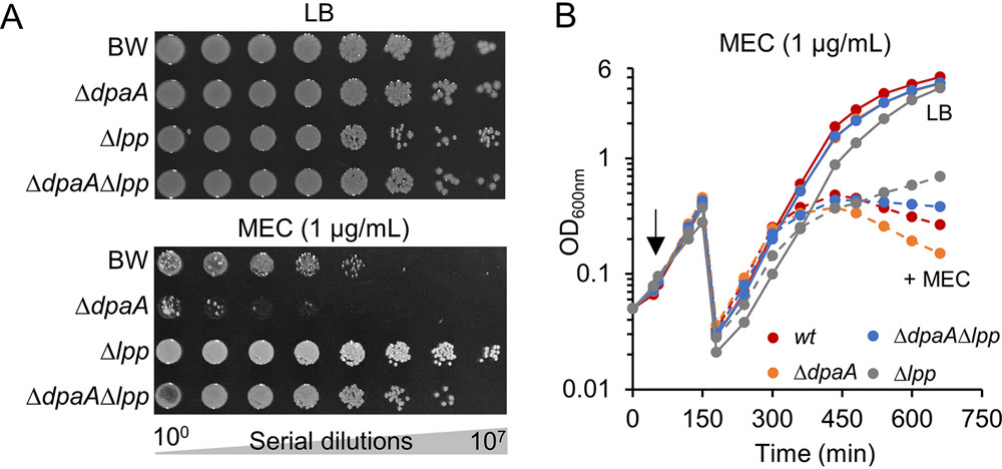
The *dpaA* mutant is more susceptible to mecillinam (MEC). (A) Serial dilutions of BW25113 and mutants (Δ*dpaA*, Δ*lpp*, or Δ*dpaA* Δ*lpp*) were spotted onto LB plates with or without mecillinam. Images were taken after incubation at 30°C for 33 h. (B) Growth of the same strains in liquid medium in the absence of mecillinam or upon addition of 1 μg/ml of mecillinam (arrow) followed by measurement of the optical density at 600 nm. Cultures were diluted 20-fold when the OD_600_ reached 0.5. Figure S4 shows that the effect is specific to mecillinam.

Our previous work showed that DpaA is important in cells that experience severe OM assembly stress. In this study, we wanted to further explore conditions under which DpaA becomes important and searched a chemical genomics database for conditions that decreased the fitness of the *dpaA* mutant strain (38). The *dpaA* mutant had negative fitness scores with the inhibitor of LPS synthesis CHIR-090 and the elongasome inhibitors A22 and mecillinam (amdinocillin), which both cause cells to become spherical (39, 40). We hypothesized that the tight connection between the OM and PG via PG-attached Lpp could complicate changes in cell shape, such as the transition of rod shape to spherical shape. To test this hypothesis, we measured the sensitivity of strains to mecillinam. When comparing the growth of BW25113, BW25113 *ΔdpaA*, BW25113 *Δlpp*, and BW25113 *ΔdpaA Δlpp* on LB agar plates supplemented with mecillinam, we observed a higher susceptibility of the *ΔdpaA* mutant (Fig. 6A). We also grew these strains in liquid LB medium in the presence or absence of 1 μg/ml of mecillinam. Cells of BW25113 *Δlpp* still grew 8.5 h after addition of mecillinam, while BW25113 *ΔdpaA Δlpp* cells showed arrested growth, and the optical density of BW25113 and BW25113 *ΔdpaA* cultures declined (Fig. 6B). Expressing *ftsQAZ* from pTB63 decreased the susceptibility to mecillinam as described previously (41), but the difference between the strains persisted. BW25113 Δ*dpaA*/pTB63 was more susceptible to mecillinam than the other strains, and expressing *dpaA* from plasmid restored mecillinam resistance (Fig. S4A). This effect was specific to mecillinam and the Δ*dpaA* strain showed similar susceptibilities to tetracycline and ampicillin, which do not cause cells to become spherical (Fig. S4B). Hence, detachment of Lpp from PG appears to be beneficial for cells that undergo radical cell shape changes.

10.1128/mBio.00836-21.4 FIG S4 Growth defect of the *dpaA* mutant is specific to amdinocillin. (A) Spot-plate assay of strains carrying the pTB63 plasmid, expressing *ftsQAZ* alone or in combination with empty plasmid (pGS100, ep) or plasmids expressing DpaA, in the presence or absence of amdinocillin. Cells overexpressing *ftsQAZ* were more susceptible to amdinocillin when they lacked *dpaA*. (B) Spot-plate assays with tetracycline (TET) or ampicillin (AMP) showed no altered sensitivity of the *dpaA* mutant. Download FIG S4, TIF file, 2.4 MB. Copyright © 2021 Winkle et al. 2021 Winkle et al. https://creativecommons.org/licenses/by/4.0/ This content is distributed under the terms of the Creative Commons Attribution 4.0 International license.

### DpaA is present in many bacteria that lack Lpp.

**FIG 7:**
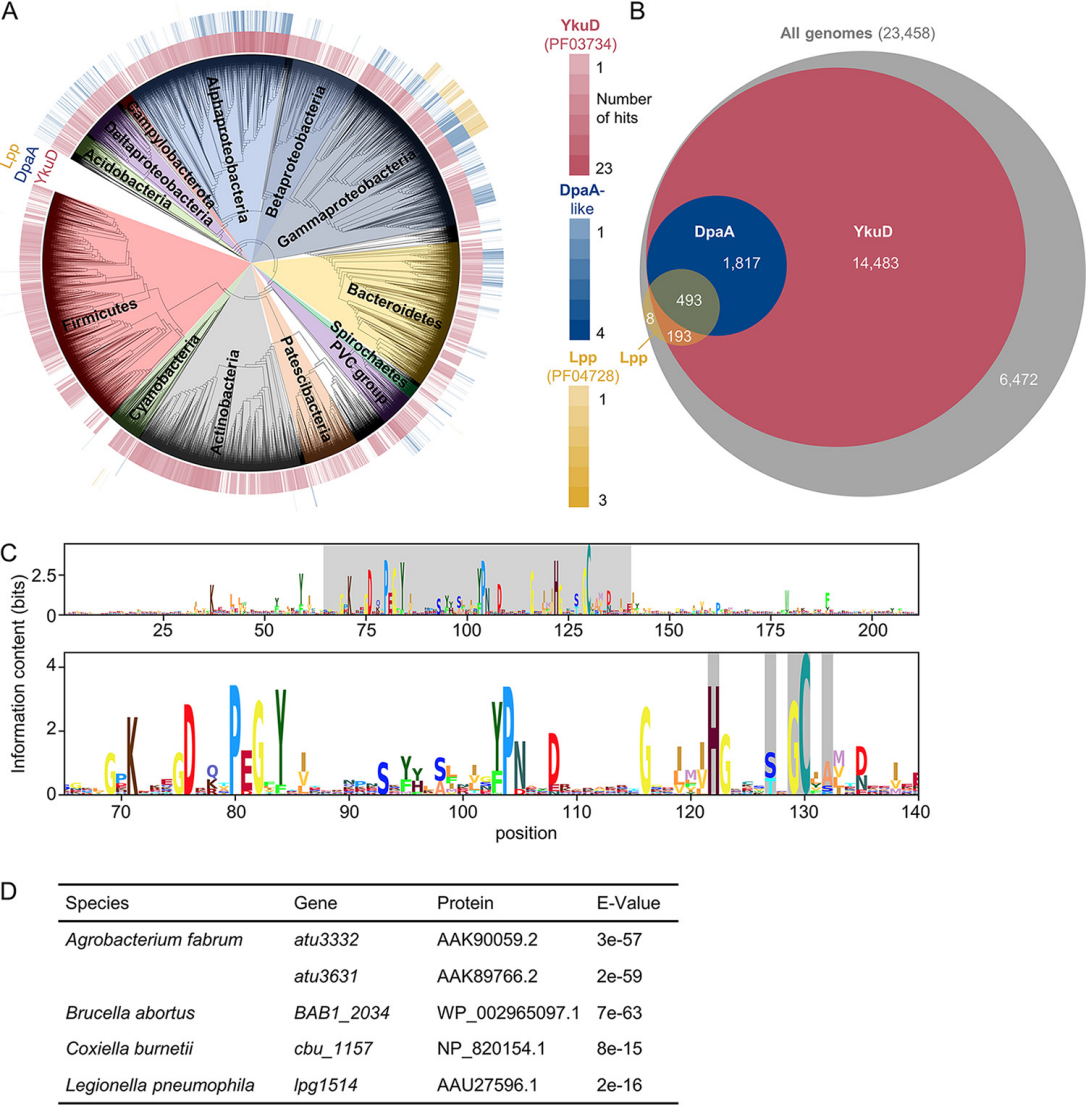
Conservation and distribution of DpaA. (A) Distribution of genes encoding proteins of the YkuD family (Pfam PF03734; red), Lpp (PF04728; yellow), and DpaA-like proteins (identified by BLAST within PF03734 proteins; blue) within the AnnoTree bacterial genome database. The intensity of the color corresponds to the number of genes identified in each genome. (B) Venn diagram showing the numbers of genomes containing the different combinations of DpaA-like (blue), Lpp (yellow) or YkuD family (red) proteins within the genomes in the database. The area of the circles is proportional to the number of genomes they represent. The gray circle indicates the total number of genomes in the database. (C) Logogram obtained from the multiple-sequence alignment of the DpaA-like proteins identified within the AnnoTree database. (Top) full alignment; (bottom) amino acid positions 65 to 140, highlighting same positions as in the alignment in Fig. 1. (D) Table showing genes encoding DpaA-like proteins identified in bacteria lacking *lpp* and known to attach OM β-barrel proteins to PG. The protein column shows NCBI protein annotation corresponding to the indicated gene; the E value column shows BLAST expected values indicating the similarity to the query sequence, E. coli DpaA.

We next asked whether DpaA co-occurs with Lpp in diderm bacteria. We analyzed the presence of proteins of the YkuD family (PF03734 in the Pfam database), Lpp-like proteins (PF04728), and DpaA-like proteins within the AnnoTree database, which contains a set of representative, fully sequenced, and consistently annotated bacterial genomes (42). YkuD-like proteins are present in 72% of the genomes, often with several copies (61,365 sequences in 16,986 genomes). Lpp-like proteins are present only within a subset of *Gammaproteobacteria* (∼3% of all genomes) (Fig. 7A and B). To identify DpaA-like proteins, we searched within the 61,365 YkuD family sequences in AnnoTree using BLAST and the E. coli DpaA sequence as a query, resulting in 2451 hits from 2,310 different genomes (Fig. 7A and B). The identified sequences are present in a wide range of diderm bacteria lacking Lpp, including species within *Alpha-*, *Gamma-*, and Deltaproteobacteria, *Acidobacteria*, and *Bacteroidetes* (Fig. 7A). About a third of the genomes encoding Lpp did not contain a DpaA-like protein, suggesting that those organisms are not capable of detaching Lpp from PG or other proteins have this function (Fig. 7B). Alignment of the sequences of DpaA-like proteins identified within AnnoTree revealed the catalytic cysteine and several conserved positions common to all YkuD-like proteins (Fig. 7C). As in E. coli DpaA (Fig. 1), most DpaA-like proteins have alanine or asparagine 2 positions after the catalytic cysteine instead of an arginine that is present in LDTs with TPase activity.

Overall, our sequence analysis suggests that species without Lpp use DpaA for a different purpose. Some of the DpaA homologues might function as ld-carboxypeptidase, consistent with their classification in the MEROPS peptidase database (43) within the C82.A01 peptidase subfamily along with the ld-carboxypeptidases Csd6 from Helicobacter pylori and Pgp2 from Campylobacter jejuni (44, 45). Alternatively, or in addition, DpaA could detach different substrates from PG, for example OMPs in *Alpha-* and *Gammaproteobacteria* (24, 25). Indeed, a search within the genomes of *Alpha-* and *Gammaproteobacteria* with PG-attached OMPs identified genes encoding DpaA-like proteins in all of them (Fig. 7D).

## DISCUSSION

**FIG 8:**
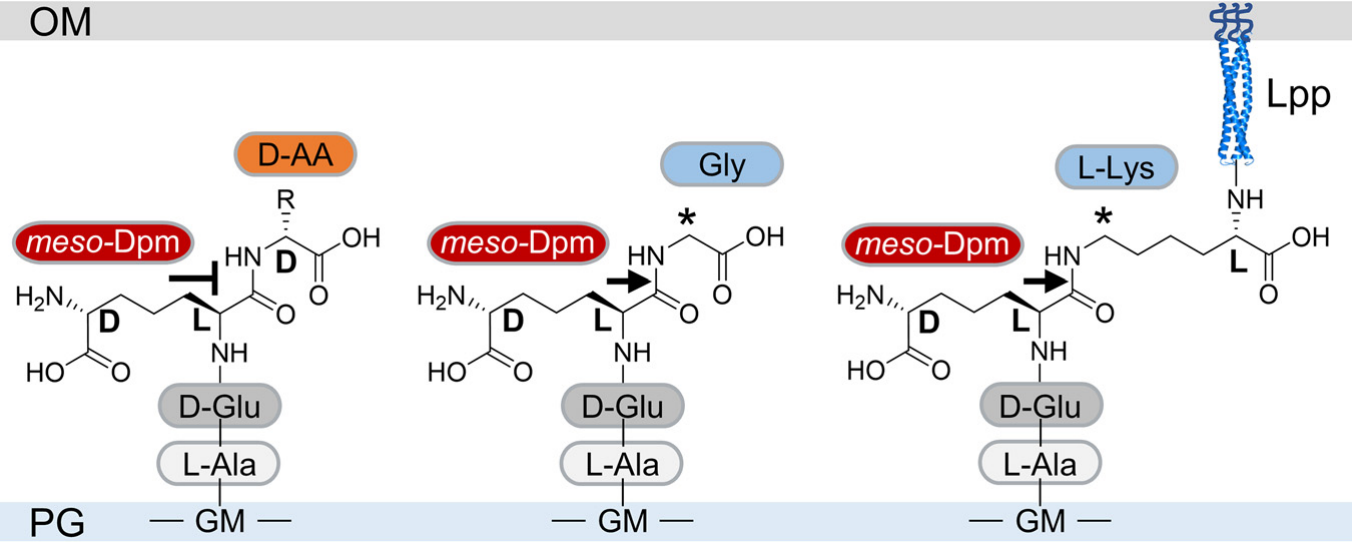
Specificity of DpaA. DpaA hydrolyzes the amide bond between the l-center of *meso-*Dpm and Gly (middle) or the l-center of *meso*-Dpm and the ε-amino group of the C-terminal l-lysine of Lpp. Both substrates have a CH_2_ group adjacent to the cleavage site (labeled with an asterisk).

DpaA is the first member of the YkuD family that does not act on ld-peptide bonds and is not a transpeptidase. Our work demonstrated that DpaA hydrolyzes the amide bond between the l-center of *meso-*Dpm in PG and the ε-amino group of Lpp (Fig. 8). Hence, the name LdtF (ld-transpeptidase F) is not appropriate anymore, and we propose to name the enzyme peptidoglycan *meso-*Dpm protein amidase A (DpaA).

Lpp is the most abundant protein in E. coli, and its attachment to PG substantially contributes to the stability of the cell envelope and prevents excessive OM vesiculation (5, 6, 9, 17). Why does the cell then have an enzyme that detaches Lpp from PG? We do not yet fully understand the importance of Lpp detachment under different conditions. Our initial data indicate that DpaA might be required under certain stress conditions, consistent with its expression profile (29). The *dpaA* mutant has reduced fitness when cells grow in the presence of mecillinam, an antibiotic that induces a stark change in cell shape (from rod shape to spherical shape) in growing cells (39). We hypothesize that under these conditions the detachment of Lpp from PG provides flexibility to the cell envelope to better coordinate the remodeling of the PG and OM during the shape change. The detachment of Lpp from PG could also be a means of controlling the amount of OM-derived vesicles during an infection, affecting the interaction of a diderm bacterium with the host organisms (46). In addition, the amount of PG-bound Lpp could affect bacterial cells within a biofilm and the outcome of bacterial competition (47). However, it remains to be studied in more detail whether DpaA affects virulence and bacterial fitness under environmental conditions or cell envelope stress.

DpaA becomes essential when the export of LPS to the outer membrane is compromised (29), presumably due to the spurious activation of peptidoglycan amidases via ActS (30). Our TraDIS data revealed another connection between LPS and DpaA. The *lapB* gene was interrupted by the transposon at a much higher frequency in the *dpaA* mutant than the wild type. Deleting *lapB* stabilizes LpxC and deregulates (enhances) LPS biosynthesis (48, 49), which causes lethality in wild-type cells but not in a hypervesiculating *lpp* mutant, which presumably releases the excess LPS into outer membrane-derived vesicles (33). The fact that transposon insertions in *lapB* are recovered at a higher frequency in the *dpaA* mutant than in the parent strain is consistent with the lower fitness of the *dpaA* mutant in the presence of CHIR-090 (34), an inhibitor of LpxC, and suggests that enhanced PG-bound Lpp (due to the absence of DpaA) has a negative effect on LPS synthesis and/or export. We hypothesize that the larger amount of PG-bound Lpp in the *dpaA* mutant hinders (but does not completely abolish) LPS export, which alleviates problems caused by excessive LPS biosynthesis. The effect of the increased amount of PG-attached Lpp points to a yet-unrecognized regulatory function of Lpp in LPS export.

Structure-based sequence alignment shows that ld-carboxypeptidases and DpaA-like proteins are closely related and that both lack the conserved arginine residue present in ld-TPases (Fig. S5). However, some residues conserved in DpaA-like proteins at the start of the catalytic domain are absent in the ld-carboxypeptidases (Fig. S5), and a BLAST search with E. coli DpaA failed to identify Csd6, while Pgp2 was identified with a high Expect value (E-value) of 1e−4 (44, 45). Consistent with this low similarity, E. coli DpaA was not able to remove terminal d-alanine residues from PG stem peptides; however, more experiments will be necessary to determine whether some DpaA-like proteins show ld-carboxypeptidase activity.

10.1128/mBio.00836-21.5 FIG S5 Sequence alignment of DpaA-like proteins and members of the YkuD family. Structure-based sequence alignment of DpaA-like proteins, ld-carboxypeptidases, and ld-transpeptidases obtained with PROMALS3D (J. Pei, B.-H. Kim, and N. V. Grishin, Nucleic Acids Res 36:2295–2300, 2008), using the structures of E. coli LdtD (PDB accession number 6NTW), Bacillus subtilis YkuD (1Y7M), and Helicobacter pylori Csd6 (4XZZ) as guides. The residues corresponding to the 4 structural loops surrounding the substrate pocket in Csd6 are highlighted in different colors. The red dots indicate the catalytic triad in Csd6, purple dots mark positions conserved in DpaA-like proteins but different in ld-carboxypeptidases, and the blue dot indicates the conserved Arg in ld-TPases but not in hydrolytic YkuD family proteins. The sequences aligned include those for E. coli DpaA (UniProt accession number P0AA99; residues 45 to 159), *Agrobacterium fabrum* ATU3332 (Q7CS68; 43 to 168) and ATU3631 (A0A3G2CVN4; 58 to 176), Brucella abortus BAB1_2034 (C4ITM3; 50 to 169), Coxiella burnetii CBU_1157 (Q83CG1; 67 to 183), Legionella pneumophila LPG1514 (Q83XL0; 72 to 186), Pseudomonas aeruginosa PA3756 (Q9HXN9; 10 to 166), Helicobacter pylori Csd6 (O25255; 73 to 181), Campylobacter jejuni Pgp2 (A7H3E2; 66 to 196), E. coli LdtD (P22525; 377 to 543), and Bacillus subtilis YkuD (O34816; 48 to 164). Download FIG S5, TIF file, 2.7 MB. Copyright © 2021 Winkle et al. 2021 Winkle et al. https://creativecommons.org/licenses/by/4.0/ This content is distributed under the terms of the Creative Commons Attribution 4.0 International license.

Depending on the growth medium, E. coli naturally contains a small proportion of stem peptides with glycine residues at position 4 (14). Other bacteria use LDTs to incorporate noncanonical d-amino acids produced by themselves or present in the environment into the same position (50, 51). Of the 4 amino acids tested, DpaA was capable of removing only glycine residues from position 4 and not the three d-amino acids tested. However, at present we cannot exclude the possibility that some bacteria employ DpaA-like enzymes to remove noncanonical d-amino acids, which are toxic when incorporated in a large amount (52). Because DpaA does not hydrolyze tetrapeptides with d-Ala at position 4, it is unlikely that DpaA-like proteins are capable of attaching d-amino acids in ld-transpeptidation reactions. The recent discovery of PG-attached OMPs in certain *Alphaproteobacteria* suggests that the DpaA-like enzymes in these species function to detach OMPs from PG (24, 25). In support of this hypothesis, the attachment of OMPs to PG occurs via N-terminal glycine or alanine, and the attachment of OM lipoprotein LimB via an internal lysine residue, producing the very same amide bonds as in TetraGly4 or in PG-bound Lpp in E. coli. Moreover, DpaA homologues are present in many bacteria, including many species that do not contain Lpp, suggesting different substrates. Hence, it remains to be tested whether DpaA-like enzymes are capable of removing OMPs from PG.

In summary, our work showed that DpaA performs a yet-unknown reaction in the bacterial cell envelope, the hydrolytic removal of a protein from PG. Presumably, DpaA provides flexibility to the bacterial cell to remodel the cell envelope when coping with certain stress situations. The discovery of this reaction also illustrates that cell envelope remodeling contributes to the robustness of bacterial cells and their lifestyles.

## MATERIALS AND METHODS

### Bacterial strains and growth conditions.

Strains used in this study are listed in Table S3. Bacteria were grown aerobically at 30°C or 37°C on LB plates or in liquid LB medium (10 g/liter of tryptone, 5 g/liter of yeast extract, 10 g/liter of NaCl; 15 g/liter of agar for plates). Antibiotics were used at the following concentrations: chloramphenicol (Cam), 25 μg/ml; kanamycin (Kan), 50 μg/ml; and tetracycline (Tet), 5 μg/ml. For monitoring cell cultures treated with mecillinam, 30°C prewarmed LB medium was inoculated with overnight cultures starting with a normalized optical density at 600 nm (OD_600_) of 0.05. The cultures were split in half at an OD_600_ of 0.1. One-half was supplemented with 1 μg/ml of mecillinam, and the other half remained untreated. The cultures were diluted 20-fold in fresh 30°C prewarmed LB supplemented or not with mecillinam at an OD_600_ of 0.5.

10.1128/mBio.00836-21.8 TABLE S3 Strains and plasmids used in this work Table S3, PDF file, 0.09 MB. Copyright © 2021 Winkle et al. 2021 Winkle et al. https://creativecommons.org/licenses/by/4.0/ This content is distributed under the terms of the Creative Commons Attribution 4.0 International license.

### Construction of E. coli deletion strains.

Deletion strains were obtained by transducing *kan*-marked alleles from the Keio E. coli single-gene knockout library (53) by P1 phage (54). The *kan* resistance cassette was removed by pCP20-encoded Flp recombinase to generate deletions with an Flp recognition target (FRT) site scar sequence (55). The removal of the *kan* gene was verified by PCR. Strains with multiple deletions were generated by sequential P1 transduction and *kan* resistance cassette removal.

### Plasmid construction.

Plasmids and oligonucleotides used in this study are shown in Tables S3 and S4, respectively. Genes were amplified by PCR using DNA from MC1061 as the template. pET28a-*ldtE* was constructed by ligase-independent cloning (56). Site-directed mutagenesis of plasmids was performed using a Q5 site-directed mutagenesis kit (New England BioLabs) following the manufacturer’s instructions.

10.1128/mBio.00836-21.9 TABLE S4 Oligonucleotides used in this study Table S4, PDF file, 0.02 MB. Copyright © 2021 Winkle et al. 2021 Winkle et al. https://creativecommons.org/licenses/by/4.0/ This content is distributed under the terms of the Creative Commons Attribution 4.0 International license.

For pET29b-DpaA-his construction, the signal sequence of *dpaA* was replaced with the one of *pelB* by a three-step PCR method (57) using pET27b as the template for *pelB*ss. The chimeric *pelB*ss-*dpaA* gene was cloned into pET29b between the NdeI and NcoI restriction sites. The thrombin cleavable sequence was inserted in the linker region between the 3′ end of the gene and the sequence codifying the His tag using the DpaA_pET29b_R primer.

### Spot plate assay.

The spot plate assays were performed as described previously (37). Serial dilutions of bacterial cultures were spotted onto LB plates supplemented with the appropriate antibiotics (Tet, 5 μg/ml; Cam, 25 μg/ml). The plates were incubated at 30°C for 33 h, and images were taken with a Syngene inGenius bioimaging system.

### PG isolation and analysis.

PG was isolated from E. coli cells and analyzed by reversed-phase HPLC as described previously (14). PG for DpaA activity assays containing bound Lpp was isolated as described previously (14) with the omission of treatments with α-amylase and pronase E.

### Purification of LdtE.

E. coli LOBSTR-BL21(DE3) cells (Kerafast) harboring the pET28a-LdtE-his plasmid were grown in 2 liters of LB autoinduction medium (LB supplemented with 0.5% glycerol, 0.05% glucose, and 0.2% lactose) for 20 h at 30°C (58). Cells were harvested by centrifugation for 15 min at 5,000 × *g* and 4°C. The cell pellet was resuspended in 100 ml of buffer I (20 mM HEPES/NaOH [pH 7.5], 1 M NaCl, 1 mM dithiothreitol [DTT], 10% glycerol) supplemented with 1 mM phenylmethylsulfonyl fluoride (Sigma-Aldrich), 1× protease inhibitor cocktail (Sigma-Aldrich), and desoxyribonuclease I (Sigma-Aldrich). Cells were broken by sonication, and insoluble cell debris was removed by ultracentrifugation for 1 h at 130,000 × *g* and 4°C. The supernatant was applied to a 5-ml HisTrap HP column equilibrated with buffer I on an ÄKTA PrimePlus. The column was washed with 20 ml of buffer I containing 40 mM imidazole, and bound protein was eluted with buffer II (20 mM HEPES/NaOH [pH 7.5], 500 mM NaCl, 1 mM DTT, 10% glycerol, 400 mM imidazole). Fractions containing LdtE were combined and dialyzed against 3 liters of buffer III [20 mM HEPES/NaOH (pH 7.5), 300 mM NaCl, 10% glycerol, 0.1 mM Tris(2-carboxyethyl)phosphine hydrochloride (TCEP)]. The sample was concentrated to 5 ml with a Vivaspin 6 filter. The protein was then further purified by size exclusion chromatography on a HiLoad 16/60 Superdex 200 (GE Healthcare) column using buffer III and a flow rate of 1 ml/min. LdtE-containing fractions were combined and stored at −80°C.

### Purification of DpaA and DpaA(C143A).

E. coli LOBSTR-BL21(DE3) (Kerafast) containing pET29b-DpaA-his [encoding PelB-DpaA(20-246)-His_6_] or pET29b-DpaA(C143A)-his [PelB-DpaA(20-246,C143A)-His_6_; cysteine 143 replaced by alanine] were grown at 25°C overnight in 4 liters of Terrific Broth (TB) autoinduction medium (59). Cells were harvested by centrifugation for 15 min at 5,000 × *g* and 14°C. The cell pellet was resuspended in 150 ml of buffer I (20 mM HEPES/NaOH [pH 7.5], 500 mM NaCl, 2 mM MgCl_2_, 10% glycerol) supplemented with 1 mM phenylmethylsulfonyl fluoride (Sigma-Aldrich), 1× protease inhibitor cocktail (Sigma-Aldrich), and DNase I (Sigma-Aldrich). Cells were broken by sonication, and the insoluble fraction was removed by ultracentrifugation for 1 h at 130,000 × *g* and 4°C. The supernatant was applied to a 5-ml HisTrap HP column preequilibrated with buffer I using an ÄKTA PrimePlus. The column was washed with 20 ml of buffer I with 40 mM imidazole, and protein was eluted in a 12-ml gradient from buffer I with 40 mM imidazole to buffer I with 400 mM imidazole. Fractions containing DpaA were combined and split into two pools. Pool 1 was dialyzed against 3 liters of buffer III (20 mM HEPES/NaOH [pH 7.5], 300 mM NaCl, 10% glycerol, 10 mM EDTA), and pool 2 was incubated with 10 U of thrombin (restriction grade; Novagen) to remove the His_6_ tag, during dialysis against 3 liters of buffer III. Proteins were further purified by size exclusion chromatography on a HiLoad 16/60 Superdex 200 (GE Healthcare) column using buffer III and a flow rate of 1 ml/min. The protein-containing fractions were aliquoted and stored at −80°C.

### Purification of LdtD.

LdtD was expressed in E. coli LOBSTR-BL21(DE3) cells harboring the overexpression plasmid pETMM82 (60) and purified as previously described (36).

### HPLC activity assay.

DpaA activity assays were carried out in a total volume of 50 μl in 20 mM HEPES/NaOH (pH 7.5) or 20 mM sodium acetate (pH 5.0) with 100 mM NaCl, 0.05% Triton X-100 (reduced), 0.1 mM TCEP, 10 μl of substrate (muropeptides or PG), and 2 μM protein. A control sample contained no protein. Purified nonreduced and desalted muropeptides (Tri-LysArg and TetraTri-LysArg) were a gift from J.-V. Höltje, Max-Planck-Institute [MPI] Tübingen, Germany. The reaction mixture was incubated overnight in a thermoshaker at 37°C and 900 rpm. The reaction was stopped by boiling the samples for 10 min at 100°C. Samples were reduced with sodium borohydride, and muropeptides were separated by reversed-phase HPLC (14), except that the gradient was 90 min instead of 135 min for samples with purified muropeptides. For activity assays on PG, the reaction products were treated overnight with the muramidase cellosyl (0.5 μg/ml) at 37°C and 900 rpm in 80 mM sodium phosphate (pH 4.8), and the released muropeptides were reduced and separated by HPLC as described above. Tri-LysArg, TetraTri-LysArg, and the products generated by DpaA from these muropeptides were collected during the HPLC run and analyzed by tandem mass spectrometry (MS/MS) as described previously (61).

### Amino acid exchange followed by DpaA reaction.

The amino acid exchange reaction with glycine, d-valine, d-glutamine, or d-lysine was performed in a final volume of 50 μl in 20 mM HEPES/NaOH (pH 7.5), 100 mM NaCl, 0.1 mM TCEP, 10 mM MgCl_2_, and 0.05% Triton X-100. Ten microliters of muropeptides obtained from PG of BW25113Δ6LDT (∼50 μg) was incubated with glycine, d-valine, d-glutamine, or d-lysine (10 mM) and LdtD (5 μM) for 6 h at 37°C. The reaction was terminated by boiling the sample for 10 min. The sample was centrifuged, and the supernatant containing the soluble muropeptides was split into two equal aliquots. In one half, the pH was adjusted to 5.0 with sodium acetate, DpaA (5 μM) was added, and the sample was incubated overnight at 37°C. The other half was treated the same way without the addition of DpaA. Samples were boiled for 10 min at 100°C and treated with sodium borohydride, and muropeptides were separated by HPLC as described above.

### DpaA activity assay against PG-Lpp.

DpaA or DpaA(C143A) (2 μM) was mixed with 10 μl of PG with attached Lpp from BW25113 *ΔdpaA* in 20 mM HEPES/NaOH (pH 7.5), 100 mM NaCl, 0.05% Triton X-100 (reduced), and 0.1 mM TCEP. Samples were incubated overnight in a thermoshaker at 900 rpm and 37°C. Soluble proteins were separated from PG by centrifugation (13,000 × *g*, 10 min, 4°C). The supernatant was collected, and proteins were separated by SDS-PAGE and stained with Coomassie brilliant blue or transferred to nitrocellulose for Western blotting and detection with an anti-Lpp antibody (kind gift from Jean-François Collet, UC Louvain, Brussels, Belgium).

### Analysis of DpaA conservation.

We used the AnnoTree database to study the conservation of DpaA-like proteins within bacteria (42). This database contains a set of archaeal and bacterial genomes which are completely sequenced and consistently annotated. The sequences of proteins identified as belonging to Pfam families PF03734 (ld-transpeptidase catalytic domain; 61,365 sequences in 16,986 genomes) and PF04728 (lipoprotein leucine zipper or Lpp; 717 sequences in 694 genomes) were downloaded from the AnnoTree website. We searched within the PF03734 sequences for DpaA-like proteins using BLAST (62) and E. coli DpaA as a query with an E value cutoff of 1E−8. This search resulted in 2,451 hits from 2,310 genomes. The number of proteins of each type per genome was counted using custom Python scripts, and the generated data sets were represented along a phylogenetic tree of all genomes in AnnoTree using iTOL (63). The sequences for all identified DpaA-like proteins were aligned using Clustal Omega (64). The resulting alignment was uploaded to the Skylign website to generate a logogram by converting to a hidden Markov model after removing mostly empty columns in the alignment (65). Next, the matrix with position frequencies was downloaded and the final logogram was generated using the logomaker Python package (66). The search for DpaA-like genes in organisms attaching β-barrel outer membrane proteins to PG was conducted using BLAST, and the genomes analyzed were those of Agrobacterium fabrum strain C58 (ASM9202v1), Brucella abortus 2308 (ASM74219v1), Coxiella burnetii RSA 493 (ASM776v2), and Legionella pneumophila subsp. *pneumophila* strain Philadelphia 1 (ASM848v1).

### TraDIS method.

Transposon mutant libraries were constructed by electrotransformation of the mini-Tn*5* transposon carrying a kanamycin resistance cassette (Epibio). Two technical replicates of each library were prepared for sequencing as described previously (32). Samples were sequenced using an Illumina MiSeq with 150-cycle v3 cartridges. Data were demultiplexed using the Fastx barcode splitter to remove the barcode unique to each sample (67). The transposon was matched and trimmed allowing for 4-bp mismatch, and surviving reads were mapped to the BW25113 reference genome using bwa mem (GenBank accession number CP009273.1). Totals of 5,645,872 and 7,579,883 reads were mapped for BW25113 and BW25113 Δ*dpaA*, respectively. Data are available for viewing at the TraDIS-vault browser: https://tradis-vault.qfab.org/. The BioTraDIS analysis package (version 1.4.5) was used to calculate the log fold change in read depth between each gene in the control and *dpaA* transposon libraries. We used a minimum of 50 reads (per gene) as the cutoff for analysis and thresholds of >2-fold change and a *q* value (*P* value adjusted for false discovery rate using the Benjamini & Hochberg method) of <0.01 (68).

### Accession number(s).

Raw data are available at the European Nucleotide Archive (ENA) under accession number PRJEB44311.
