# Supplementary figures and images for: DpaA Detaches Braun’s Lipoprotein from Peptidoglycan
Source: mBio. 2021 May 4;12(3):e00836-21. doi: 10.1128/mBio.00836-21 (PMC8263019; doi:10.1128/mBio.00836-21)

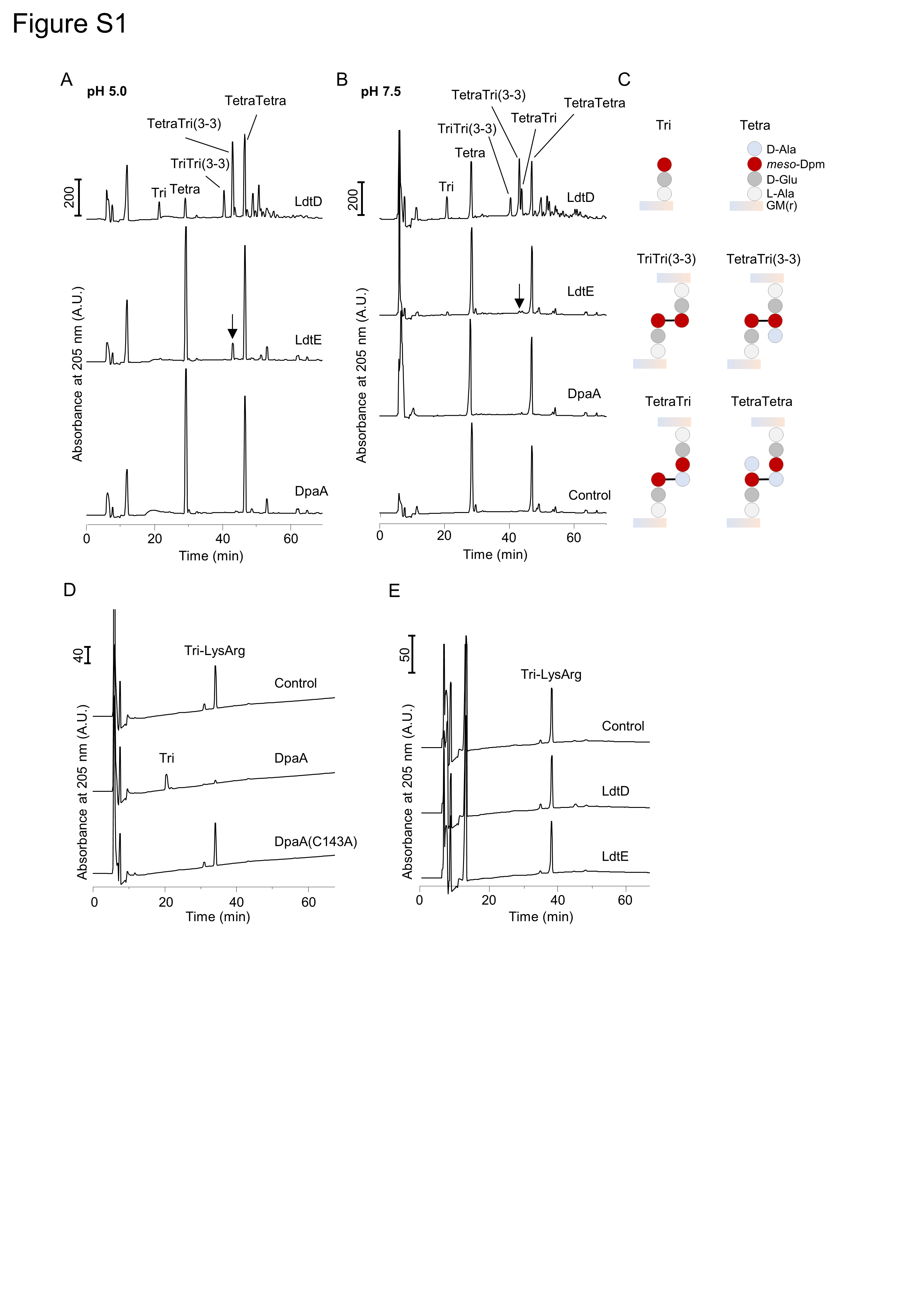

Supplement: FIG S1 [file mbio.00836-21-sf001.tif]

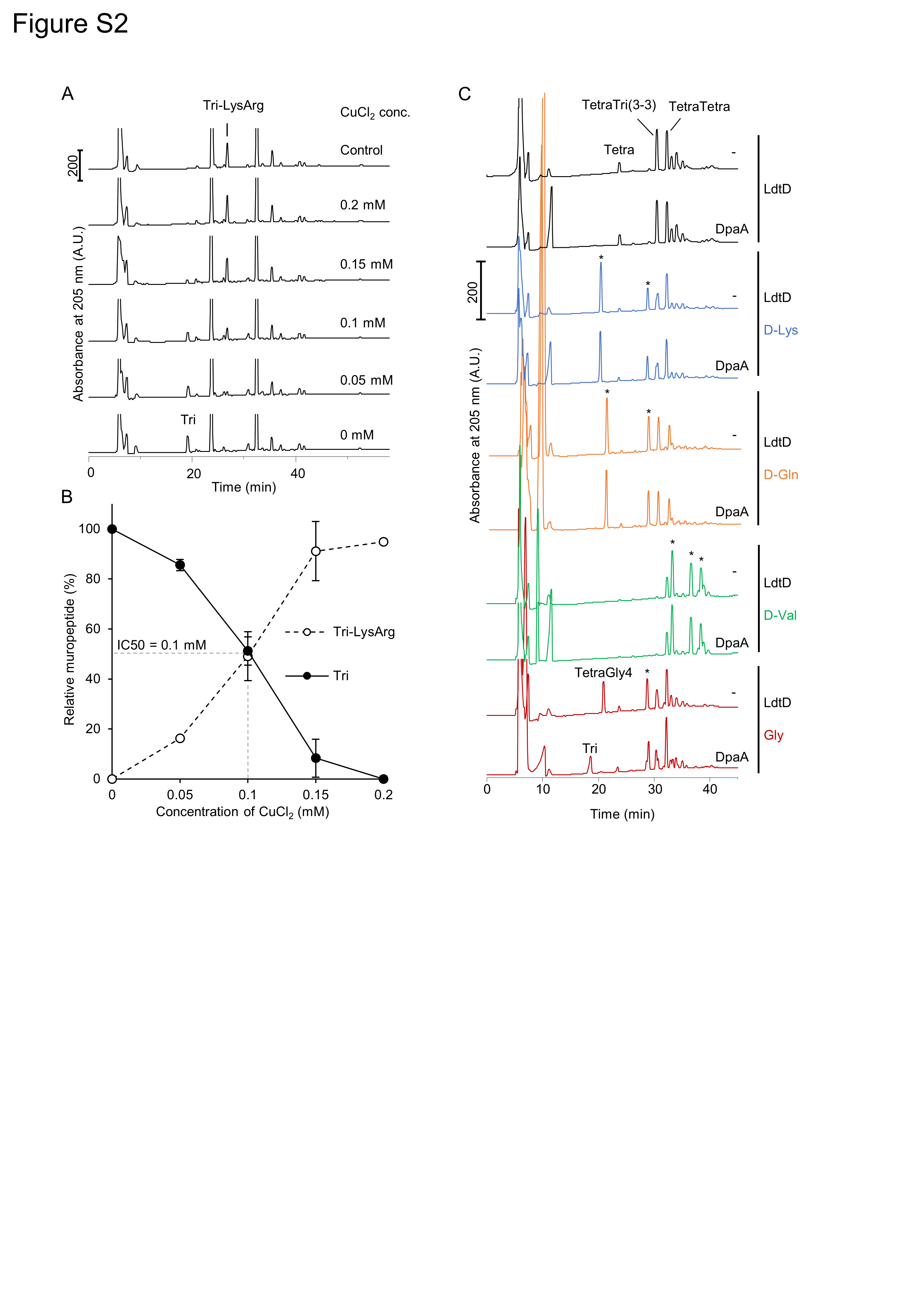

Supplement: FIG S2 [file mbio.00836-21-sf002.tif]

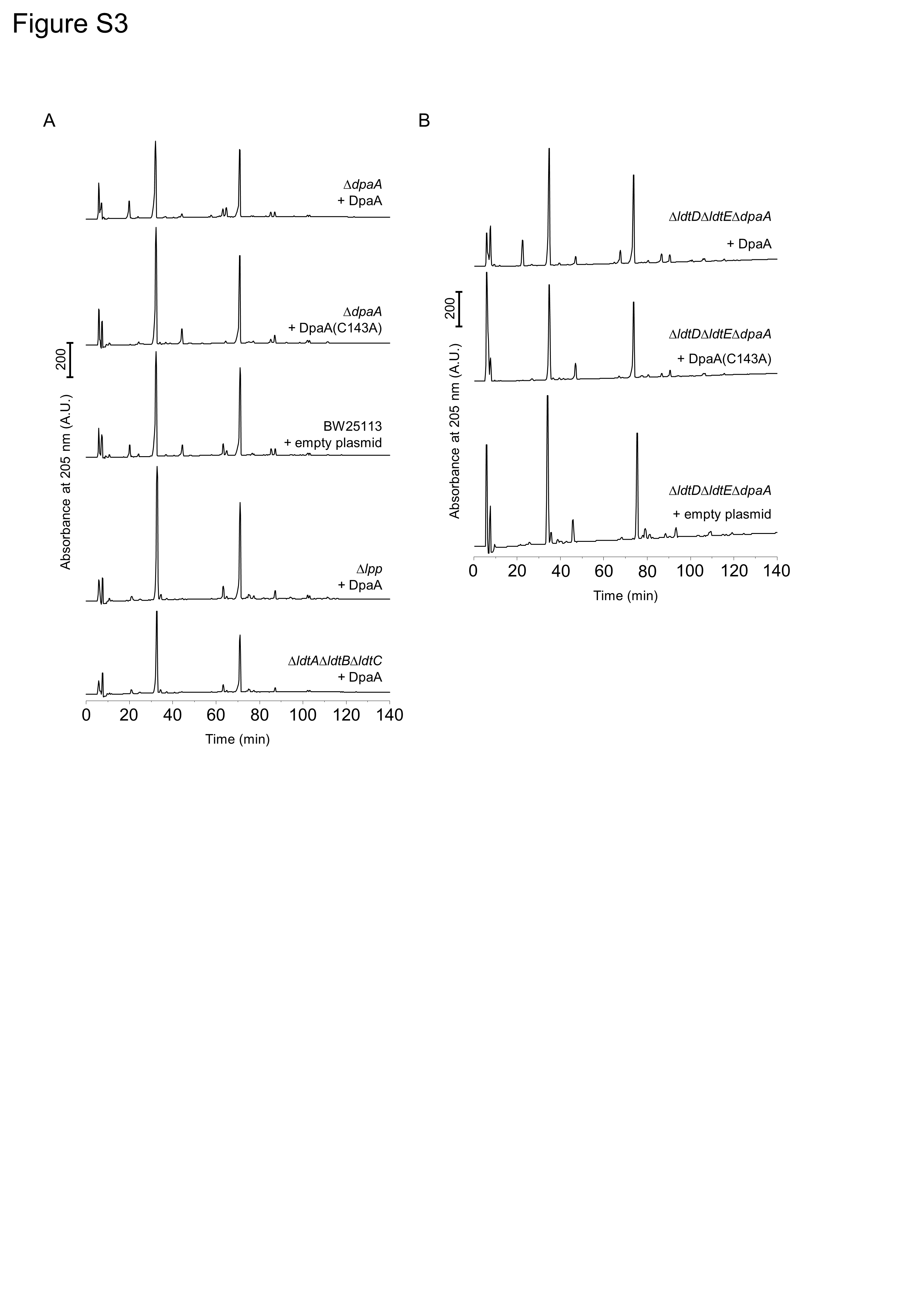

Supplement: FIG S3 [file mbio.00836-21-sf003.tif]

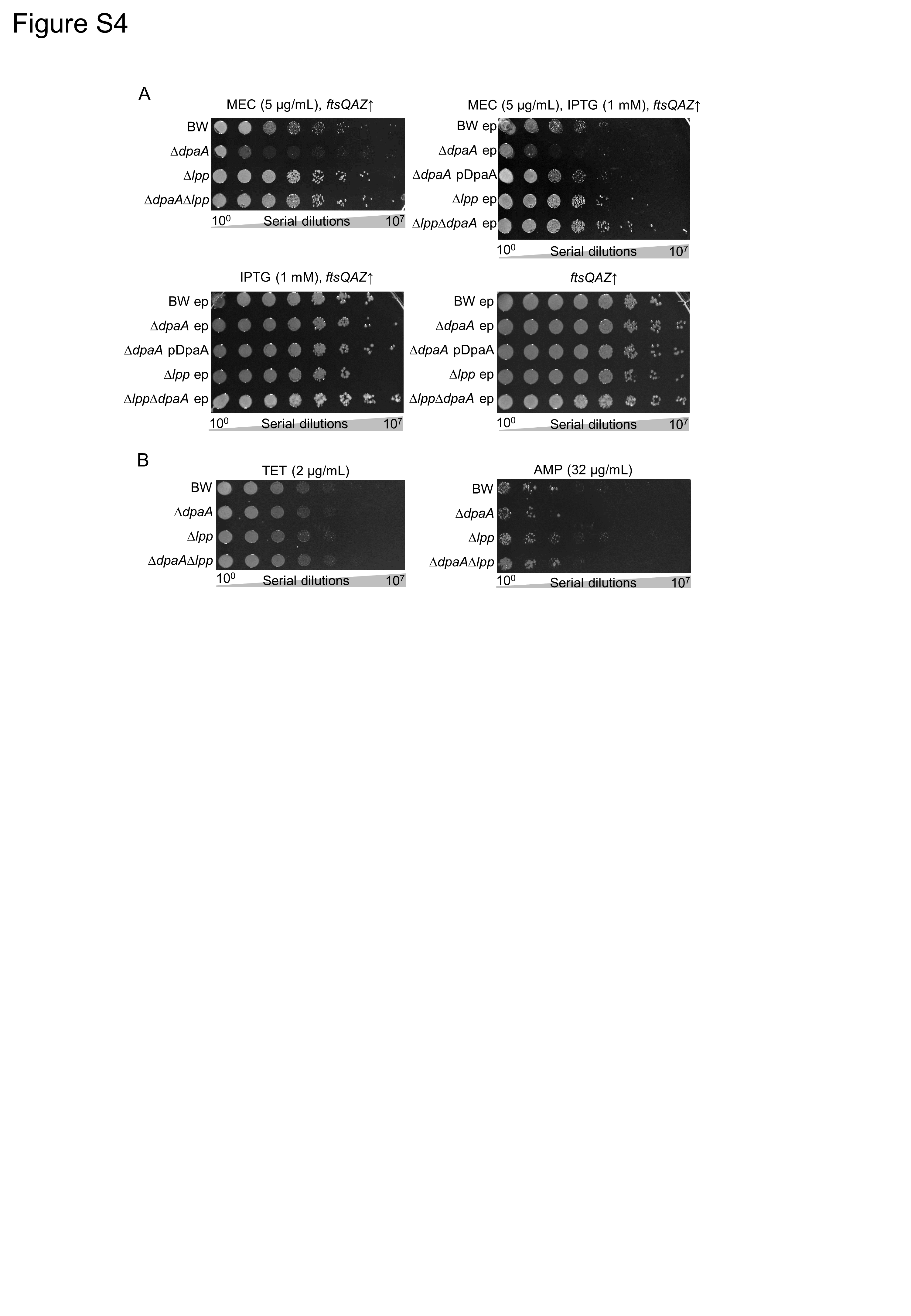

Supplement: FIG S4 [file mbio.00836-21-sf004.tif]

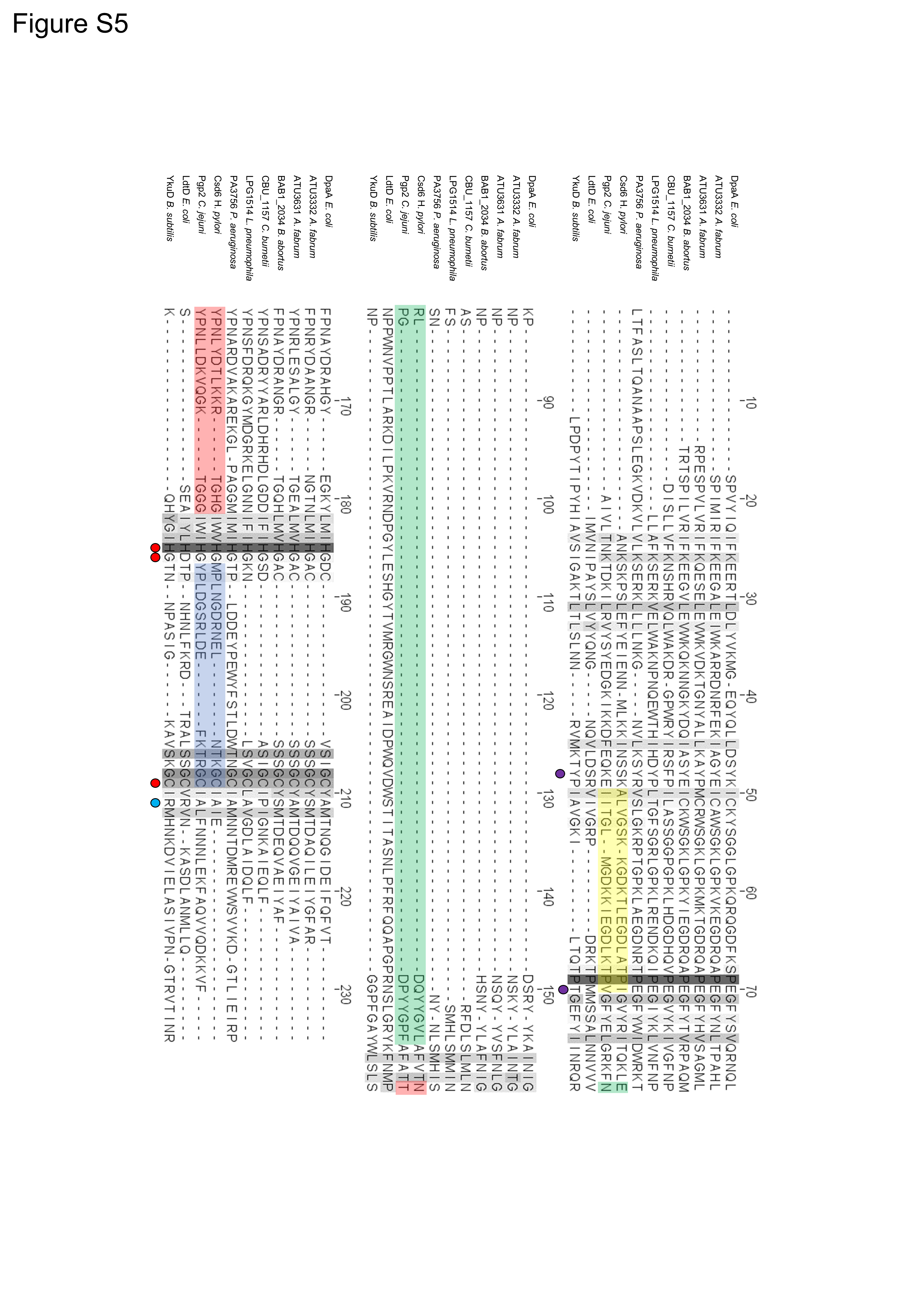

Supplement: FIG S5 [file mbio.00836-21-sf005.tif]
