# Supplementary material for: DpaA Detaches Braun’s Lipoprotein from Peptidoglycan
Source: mBio. 2021 May 4;12(3):e00836-21. doi: 10.1128/mBio.00836-21 (PMC8263019; doi:10.1128/mBio.00836-21)
Supplement: TABLE S2 [file mbio.00836-21-st002.pdf]

Table S2. MS analysis of collected muropeptide fractions (**Fig. 3**).

Muropepide (reduced)	Retention time (min)	Measured mass (charge) (Da)	Measured neutral mass (Da)	Theoretical neutral mass (Da)
**Tri**	20.2	870.8614 (+1)	869.8536	870.3706
**Tri-LysArg**	34.1	578.1715 (+2)	1154.3274	1154.5667
**TetraTri**	42.4	897.4247 (+2)	1792.8338	1793.7677
**TetraTri-LysArg**	51.2	693.4853 (+3)	2077.4325	2077.9638
