# Supplementary material for: DpaA Detaches Braun’s Lipoprotein from Peptidoglycan
Source: mBio. 2021 May 4;12(3):e00836-21. doi: 10.1128/mBio.00836-21 (PMC8263019; doi:10.1128/mBio.00836-21)
Supplement: TABLE S3 [file mbio.00836-21-st003.pdf]

Table S3. Strains and plasmids used in this work.

Strains / plasmids	Relevant features	Reference / source
Strains		
BL21(DE3)	Overexpression strain F- *ompT, hsdSB*(rB-mB-) (p_{lac}UV5::T7*gene*1), λ(DE3)	Novagen
LOBSTR BL21(DE3)	F– *ompT hsdS*B(rB– mB–) *gal dcm* (DE3), carries genomically modified copies of *arnA* and *slyD*	Kerafast
BW25113	*lacI^q^* $rrnB_{T14}$ Δ$lacZ_{WJ16}$ *hsdR514* Δ$araBAD_{AH33}$ Δ$rhaBAD_{LD78}$	(1)
BW25113Δ*lpp*	BW25113Δ*lpp::*frt	This work
(AMM14) BW25113Δ*ldtABC*	BW25113Δ*ldtA::*frt Δ*ldtB::*frt Δ*ldtC::*frt	(2)
(AMM28) BW25113Δ*ldtDE*Δ*dpaA*	BW25113Δ*ldtD::*frt Δ*ldtE::*frt Δ*dpaA::*frt	(2)
BW25113Δ6LDT	$lacI^q$ $rrnB_{T14}$ Δ$lacZ_{WJ16}$ *hsdR514* Δ$araBAD_{AH33}$ Δ$rhaBAD_{LD78}$Δ*ldtA* Δ*ldtB* Δ*ldtC* Δ*ldtD* Δ*ldtE* Δ*dpaA*	(3)
(AMM24) BW25113Δ*dpaA*	BW25113Δ*dpaA::*frt	(4)
BW25113Δ*dpaA*Δ*lpp*	BW25113Δ*dpaA::*frt Δ*lpp::*frt	This work
(AMM30) *araB*p*lptC* Δ*dpaA*	BW25113 Φ(*kan araC araB*p*lptC*)1 Δ*dpaA::*frt	(4)
Plasmids		
pETMM82	pETMM82 derivative; expresses LdtD with an N-terminal DsbC and a 6×His tag	(5)
pET28a-LdtE-his	$LdtE^{1\text{-}334}$-His_6, native signal peptide, Kan^R	This work
pET29b-DpaA-his	PelBss- $DpaA^{20\text{-}246}$-His_6, Kan^R	This work
pET29b-DpaA(C143A)-his	PelBss- $DpaA^{20\text{-}246}$-His_6 C143A, Kan^R	This work
pGS100	pGZ119EH derivative, contains TIR sequence downstream of p*tac*, Cam^R	(6)
pGS124 (pDpaA)	pGZ119H derivative; expresses DpaA under the *tac* promoter; Cam^R	(4)
pGS124-DpaA(C143A) pDpaA(C143A)	pGZ119H derivative; expresses DpaA DpaA(C143A), *tac* promoter; Cam^R	This work
pTB63	*tetA* PftsQAZ::*ftsQAZ*	(7)
pCP20	FLP expression, temperature sensitive replication; Cam^R and Amp^R.	(1)

References:

1. Datsenko KA, Wanner BL. 2000. One-step inactivation of chromosomal genes in *Escherichia coli* K-12 using PCR products. *Proc Natl Acad Sci U S A* 97:6640-5.

2. Peters K, Pazos M, Edoo Z, Hugonnet JE, Martorana AM, Polissi A, VanNieuwenhze MS, Arthur M, Vollmer W. 2018. Copper inhibits peptidoglycan LD-transpeptidases suppressing beta-lactam resistance due to bypass of penicillin-binding proteins. Proc *Natl Acad Sci U S A* 115:10786-10791.

3. Kuru E, Lambert C, Rittichier J, Till R, Ducret A, Derouaux A, Gray J, Biboy J, Vollmer W, VanNieuwenhze M, Brun YV, Sockett RE. 2017. Fluorescent D-amino-acids reveal bi-cellular cell wall modifications important for *Bdellovibrio bacteriovorus* predation. *Nat Microbiol* 2:1648-1657.

4. Moré N, Martorana AM, Biboy J, Otten C, Winkle M, Serrano CKG, Monton Silva A, Atkinson L, Yau H, Breukink E, den Blaauwen T, Vollmer W, Polissi A. 2019. Peptidoglycan remodeling enables *Escherichia coli* to survive severe outer membrane assembly defect. *mBio* 10(1):e02729-18.

5. Hugonnet JE, Mengin-Lecreulx D, Monton A, den Blaauwen T, Carbonnelle E, Veckerle C, Brun YV, van Nieuwenhze M, Bouchier C, Tu K, Rice LB, Arthur M. 2016. Factors essential for L,D-transpeptidase-mediated peptidoglycan cross-linking and beta-lactam resistance in *Escherichia coli*. *Elife* 5:e19469.

6. Sperandeo P, Pozzi C, Deho G, Polissi A. 2006. Non-essential KDO biosynthesis and new essential cell envelope biogenesis genes in the *Escherichia coli yrbG-yhbG* locus. *Res Microbiol* 157:547-58.

7. Bendezu FO, Hale CA, Bernhardt TG, de Boer PA. 2009. RodZ (YfgA) is required for proper assembly of the MreB actin cytoskeleton and cell shape in E. coli. *EMBO J* 28:193-204.
