# Supplementary material for: DpaA Detaches Braun’s Lipoprotein from Peptidoglycan
Source: mBio. 2021 May 4;12(3):e00836-21. doi: 10.1128/mBio.00836-21 (PMC8263019; doi:10.1128/mBio.00836-21)
Supplement: TABLE S4 [file mbio.00836-21-st004.pdf]

Table S4. Oligonucleotides used in this study.

Primer	Sequence 5'→3'	Construct(s)
DpaAGS_C143A_F	TTC CAT CGG CGC CTA CGC AAT GAC	pGS124-DpaA(C143A)
DpaAGS_C143A_R	ACA CAA TCG CCG TGG ATC	pGS124-DpaA(C143A)
DpaA_C143A_F	TCATTGCGTAggcGCCGATGGAAAC	pET29b-DpaA(C143A)-his
DpaA_C143A_R	CCAATCAGGGTATTGATG	pET29b-DpaA(C143A)-his
LdtE_pET28a_F	TTA GTT CCT CGT GGT TCT CTC GAG CAC CAC CAC CAC	pET28a-LdtE-his
LdtE_pET28a_R	CCA TGG TAT ATC TCC TTC TTA AAG TTA AAC AAA ATT	pET28a-LdtE-his
LdtE_Insert_F	GAA GAA GAT ATA CCA TGG ATG AAA CGC GCG TCT TTG	pET28a-LdtE-his
LdtE_Insert_R	AGA ACC ACG AGG AAC TAA CTG CGT CAC GCG TAA CAT	pET28a-LdtE-his
PelBss-DpaA_pET29b_F	GCGCC CAG CCG GCG ATG GCc ggt ttg ctg ggc agc ag	pET29b-DpaA-his
DpaA_pET29b_R	GGA TCC GAT ATC GCC ATG GAA CCG CGT GGC ACC AGg gta cct ttt gcc tcg ggg agc gtg	pET29b-DpaA-his
PelBss_F	GGC CAT CGC CGG CTG GGC	pET29b-DpaA-his
PelBss_R	ACT ATA GGG GAA TTG TGA GCG G	pET29b-DpaA-his
